# Sustained Effects of Government Response on the COVID-19 Infection Rate in China: A Multiple Mediation Analysis

**DOI:** 10.3390/ijerph182312422

**Published:** 2021-11-25

**Authors:** Taixiang Duan, Zhonggen Sun, Guoqing Shi

**Affiliations:** 1 Department of Sociology, Hohai University, Nanjing 211100, China; duantaixiang@163.com; 2 School of Public Administration, Hohai University, Nanjing 211100, China; sunzhonggen@hhu.edu.cn; 3 Asian Research Center, Hohai University, 8 Focheng West Road, Jinagning District, Nanjing 211100, China

**Keywords:** sustained effects, government response, infection rate, risk perception, adoption of PARs, COVID-19, China

## Abstract

Many scholars have considered the relationship between the government response to COVID-19, an important social intervention strategy, and the COVID-19 infection rate. However, few have examined the sustained impact of an early government response on the COVID-19 infection rate. The current paper fills this gap by investigating a national survey performed in February 2020 and infection data from Chinese cities surveyed 1.5 years after the outbreak of COVID-19. The results suggest that the Chinese government’s early response to COVID-19 significantly and sustainedly reduced China’s COVID-19 infection rate, and that this impact worked through risk perception, the adoption of protective action recommendations (PARs), and the chain-mediating effects of risk perception and the adoption of PARs, respectively. These findings have important practical value. In demonstrating how government response and infection rate at the macro level are connected to the behaviour of individuals at the micro level, they suggest feasible directions for curbing the spread of diseases such as COVID-19. When facing such public health emergencies, the focus should be on increasing the public’s risk perception and adoption of PARs.

## 1. Introduction

COVID-19 is caused by a novel coronavirus even more infectious than the virus responsible for the 2002–2004 SARS outbreak. COVID-19 was classified by the World Health Organization as a pandemic on March 11, 2020 [1]. By August 5, 2021, 200 million confirmed cases and 4.26 million deaths had been reported worldwide. However, China had reported only 121,326 confirmed cases and 5651 deaths [2]. These figures are surprisingly low, given our limited understanding of the virus and the absence of effective drug treatments. At the time of writing, only 36,798 new cases of infection have been reported in China in the last year. Why are the numbers of infections and deaths in China so much lower than those in other countries? A research team from the University of Oxford shed light on this question by reporting a link between government response and the spread of COVID-19, with strong early intervention by the Chinese government playing a crucial role in limiting the spread of the disease [3]. Scholars have generally agreed that the Chinese government’s early intervention was very effective [4,5,6,7,8,9,10,11]. However, the government relaxed its intervention efforts in May 2020, when the world considered China to be at the highest risk of experiencing a sustained COVID-19 epidemic, and there have since been no major COVID-19 spikes in China. Did the government’s early intervention thus have a sustained impact on COVID-19 infection, limiting the later spread of the disease? If so, what was the mechanism of this impact? These questions have not been explored in previous studies, but answering them may help to curb the future spread of a pandemic such as COVID-19. In the current paper, we attempt to fill this research gap.

## 2. The Effect of Government Response on the Infection Rate

### 2.1. Immediate Effects of Government Response on the Infection Rate

Studies have shown that social interventions are needed to control the spread of epidemic diseases. Bauch and Galvani point out thatcontrol of the SARS coronavirus depended partly on the degree of acceptance of quarantine and isolation among the population; such acceptance is often determined by social norms [12]. In the book *The Rules of Contagion: Why Things Spread and Why They Stop*, Kucharski concluded that the factors that influence the reproduction number of an epidemic disease include duration, opportunities, transmission, and susceptibility [13]. In his view, curing a patient reduces the duration of infection, isolating a patient reduces the opportunities for infection, wearing a condom or mask reduces contagion, and vaccination reduces population susceptibility. Government response to COVID-19 consists of social interventions implemented to curb the spread of the disease. COVID-19 intervention policies are complex and vary between countries, but they can be broadly categorised into five major areas, namely, containment and closure, economic responses, health systems, vaccine policies, and miscellaneous policies [3]. Many studies have attempted to determine the most effective intervention policies. For example, Richard et al. examined the effects of four types of government response—event bans, school closures, bar and pub closures, and lockdown—and discovered that event bans and school closures directly reduced virus transmission, while the influence of a full lockdown was slightly delayed [14]. Scholars have used epidemiological data on COVID-19 and anonymised migration data to simulate outbreaks and intervention effects across China. A comparison of infections in Wuhan, Hubei province, with those in other cities in Hubei and cities in other provinces revealed that early detection and isolation were more effective than travel restrictions. Reducing social contact curbed the spread of COVID-19 and prevented or delayed the arrival of a second wave of the outbreak. The authors also found that although travel restrictions had not prevented the virus from spreading from Wuhan, they had prevented its wider geographical spread [4].

The degree of policy implementation is also an important predictor of the COVID-19 infection rate. An international comparative study using data from China, Italy, Brazil, Canada, the United Kingdom, and the United States found that the stringency of intervention policy implementation was negatively associated with the number of new cases. This study also found that the Chinese government had maintained strong prevention and control measures for the first 100 days of the outbreak, during which China had experienced a dramatic decrease in infections [3]. When the virus was first detected in Guangdong province, the province’s health commission quickly activated a Level I emergency response and implemented a series of public interventions, including traffic restrictions, social distancing, home and centralised quarantines, medical resource mobilisation, and other prevention and control measures, which significantly restrained the local spread of the disease [15]. Differences in the degree of policy implementation may stem from differing individual responses to government policies or from differences in national policy environments, such as social norms, cultural traditions, the political atmosphere, and other macro-level factors that interact with government response [16,17]. In general, policies that are strictly enforced tend to bring about better results, especially in the early stages [3,4,6,14,15,16].

The timing of policy initiation is another important predictor of the effectiveness of government response to COVID-19. Take social distancing as an example. A series of studies found that isolating infected people decreased and delayed transmission as well as reducing the epidemic’s peak [4,8,18,19,20]. Using counterfactual simulations, another study discovered that if the same restrictions on mobility had been implemented just one to two weeks earlier, a substantial number of cases and deaths would have been avoided. Specifically, 61.6% of the infections and 55% of the deaths reported nationwide by May 3, 2020 could have been avoided if these preventive and control measures had been implemented just one week earlier [4]. A study of the relationship between the first emergency quarantine policy in Portugal from 18 March to 2 May 2020 and the public’s health behaviour showed that 79.8% of the participants, whose physical activity took place indoors, complied with the government quarantine measures and adapted their health behaviour [21]. Therefore, early social distancing plays a key role in relieving pressure on healthcare facilities and ensuring a sustained supply of healthcare resources. At this level, the timing of social distancing implementation is crucial to controlling large-scale outbreaks. Social distancing has been shown to reduce not only new cases but also cumulative cases. This implies that early government intervention may have some sustained effects, in that people became more aware of the virus during home isolation and were more likely to adopt protective action recommendations (PARs) after home isolation, thereby reducing their own infection rates.

### 2.2. Sustained Effects of Government Response on Infection Rate

The findings of the aforementioned studies demonstrate the immediate inhibitory impact of government response on the spread of COVID-19. However, little is known about the sustained effects of an early government response on the COVID-19 infection rate. We propose that there are two ways in which government response can exert a sustained impact on the spread of infection: one is by influencing individuals psychologically, such as through risk perception, precautionary awareness, emotions, and confidence; and the other is by directly influencing individuals’ protective behaviours, such as mask-wearing and social distancing.

#### 2.2.1. The Mediating Role of Risk Perception

Risk perception, a core concept of the risk society, has received much attention from researchers, especially in the context of the COVID-19 pandemic [22,23,24]. Risk perception is an individual’s subjective judgement of the characteristics and severity of risk, and it influences their decision-making when faced with an unexpected, uncontrollable, unknown, and potentially fatal public crisis such as COVID-19 [22]. A large body of research suggests that risk perception can be a powerful mediator of the relationship between social intervention measures and the spread of disease [22,25,26].

First, government response has a significant impact on individuals’ risk perception. Studies have established that providing detailed information on government response to COVID-19, especially positive messages about infection risk prevention and control [22,26,27,28,29,30,31,32,33], such as news of the construction of the Fangcang shelter hospital and the preventative efforts and achievements of health workers and volunteers, as well as protection guidelines and other information about COVID-19, can influence people’s perception of risk and promote their cooperation with epidemic prevention, thereby reducing the COVID-19 infection rate.

Second, government response can alleviate the impact of negative emotions on the COVID-19 infection rate by altering risk perception. In the early stages of an epidemic, the public may hold conflicting attitudes towards and perceptions of the severity of the threat posed by the unknown disease; some may be positive and optimistic, while others may be negative and pessimistic. Research has found that risk perceptions based on positive emotions, such as gratitude and hope, are critical to government efforts to promote cooperation to prevent the spread of an infectious disease [34]. Conversely, risk perceptions based on negative emotions, such as anxiety and fear, can reduce individuals’ cooperation with government efforts [35]. Health anxiety, measured on a continuum from no health anxiety to pathological health anxiety, can also influence individuals’ cooperation with the government to prevent the spread of an epidemic [36,37]. Studies have shown that information and advice released by the government can lead to the formation of appropriate risk perceptions [22,26], which can alleviate negative emotions [29,38,39,40]. Therefore, we can infer that risk perception mediates the impact of government response on infection rate

#### 2.2.2. The Mediating Role of PAR Adoption

During a pandemic, even when governments have developed early intervention policies, the cooperation of individuals is necessary to stop the spread of the disease. Studies have found that PAR adoption by individuals is an extremely important strategy for interrupting the spread of the COVID-19 pandemic [22]. Government response can influence whether individuals adopt PARs. When people see the authorities taking swift action, they are more likely to take the threat seriously and thus to comply with prevention measures. In addition, legal disciplinary mechanisms, cultural norms, and public opinion can lead individuals to comply passively with PARs to avoid possible punishment and/or public condemnation, thus reducing their likelihood of being infected. During the Chinese New Year festival in 2020, the Chinese government called for strict home isolation for all residents to stop the spread of COVID-19, which to some extent created a new social norm. The policy was conveyed to communities through announcements and brochures on the importance of home isolation. Volunteers and property staff monitored residents’ observance of the policy, which directly increased their awareness of and compliance with PARs. As a result, the spread of COVID-19 was effectively controlled [22].

However, the effectiveness of government response in reducing infection rate through individuals’ adoption of PARs can vary between individuals. For example, some scholars have found that people with higher levels of perceived distress during the outbreak have more public health knowledge and are therefore more likely to adopt PARs, while people with lower levels of perceived distress know less about public health and are thus less likely to adopt PARs [40,41,42]. Scholars have also found a correlation between an individual’s perception of the probability of infection and their adoption of PARs during a pandemic; when individuals perceive the probability to be higher, they are more likely to adopt PARs to reduce the risk of infection or prevent its occurrence [40,41,43,44]. Using a protective action decision model, Lindell and Perry found that individuals’ psychological risk perception and protective behaviours were shaped by their attention to the information disclosed by society and the environment [45]. Although strict interventions lock down local communities and disrupt normal social interactions, they also enhance people’s sense of efficacy in preventing infection in their communities. People with higher levels of efficacy, such as healthcare professionals, are more likely to adopt PARs and cooperate with the government’s intervention policies. In contrast, people with lower levels of efficacy, such as those who perceive the government’s response to be ineffective, are less inclined to cooperate, thus doing little to limit the spread of the pandemic [22,38].

#### 2.2.3. The Multiple Mediating Effects of Risk Perception and PAR Adoption

Studies have found that risk perception is an important factor in the decision to adopt PARs [22,26]. Individuals with lower levels of risk perception tend to be less vigilant in guarding against infection, which may reduce their likelihood of PAR adoption and in turn increase the infection rate [46]. Two characteristics of COVID-19 risk perception, perceptions of the pandemic’s severity and feelings of anxiety, are significantly associated with individuals’ COVID-19 PAR adoption. Researchers have found that people who perceive the pandemic as more severe are more likely to collect information about it and follow various government protection strategies, increasing their confidence in adopting and thus their likelihood of adopting PARs. Conversely, individuals who perceive the pandemic as less threatening and feel less anxious about it are less likely to take protective measures [36,37,38,39,47,48].

The social amplification of risk framework proposed by Kasperson and colleagues argues that the social context in which government intervention is implemented, including the interaction effects between crisis events and individual psychology, institutional culture, and social norms, can impact individual risk perceptions [49]. For example, government policies and social norms supporting public mask-wearing and international travel control can influence individual risk perceptions and effectively reduce COVID-19 mortality. Therefore, the government, as the main body responsible for pandemic management, formulates and implements intervention policies, including various public initiatives such as government-organised rescue and treatment, publicity, and prevention and control, which change the social environment, affect people’s risk perceptions, and subsequently influence their PAR adoption decisions [22]. Researchers have found that per capita COVID-19 mortality is lower in countries with cultural norms or government policies supporting public mask-wearing [17]. Studies have examined the relationship between government response, risk perception, and PAR adoption and determined that risk perception is an important mediator between government response and PAR adoption [22]. Thus, risk perception and PAR adoption are not independent factors affecting infection rate. Government response may affect infection rate by influencing the public’s risk perception and therefore promoting public compliance with protective behaviours.

**Figure 1:**
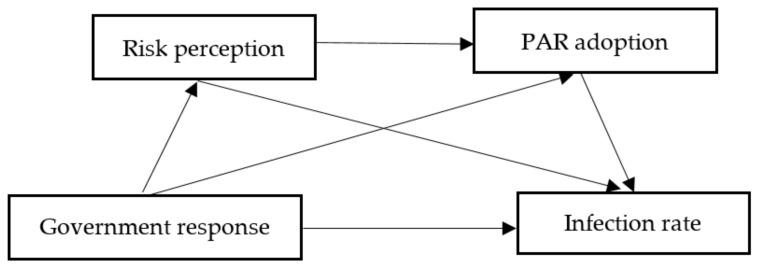
Conceptual model.

Therefore, this study investigates the sustained effects of government response on the COVID-19 infection rate in China. We propose a conceptual model of government response, risk perception, PAR adoption, and infection rate based on the literature, as shown in Figure 1, to examine the mediational pathway between government response and infection rate. We posit the following three hypotheses:

**Hypothesis** **1(H1).** *Risk perception mediates the association between government response and infection rate.*

**Hypothesis** **2(H2).** *PAR adoption mediates the association between government response and infection rate.*

**Hypothesis** **3(H3).** *The relationship between government response and infection rate is sequentially mediated by risk perception and PAR adoption.*

## 3. Materials and Methods

### 3.1. Participants and Procedure

The data for the present study were drawn from a large-scale research project conducted between 11 and 18 February 2020 by the School of Public Administration of Hohai University that investigated the psychosocial impact of COVID-19 on the public in China. The project distributed questionnaires via the Internet and conducted a survey using quota sampling. It collected 8000 questionnaires in 13 prefecture-level cities in Jiangsu province and another 30 provincial capitals in mainland China. Before beginning the survey, the participants were informed that their participation was voluntary and could be discontinued at any time. They were also informed that no personal information would be collected; their survey responses would remain anonymous and have no bearing on their academic standing. The project was approved by the Human Subjects Ethics Sub-Committee at the university with which the corresponding author is affiliated.

Originally, 8138 people completed the survey. After eliminating the survey responses of participants younger than 18 and questionnaires with many missing values, a total of 7092 valid samples were ultimately obtained. Infection rate was calculated based on the numbers of confirmed cases published by local health committees and the official 2020 population data for the cities surveyed.

### 3.2. Measures

Infection rate: ‘Infection rate’ refers to the number of confirmed cases over the past year per 100,000 population. We collected the number of confirmed COVID-19 cases announced by the health commission of each surveyed city between February 2020 and February 2021 and the permanent population data in the statistical yearbooks of each city for 2020. We then calculated each city’s infection rate based on these data.

Government response: ‘Government response’ refers to the actions taken by the government to advise or mandate that the public and private sectors take certain measures to restrict the severity or spread of the pandemic. Based on the ‘Level I Response Measures for Pneumonia Outbreak in Response to Novel Coronavirus Infection’ issued by each province, the research team compiled a list of 20 common prevention and control measures (see Table 1). The respondents were asked in the questionnaire whether their local governments had adopted these measures. If a measure had been adopted, the response was recorded as ‘1′ and ‘0′ otherwise. The sum was divided by 20 to calculate the government response index.

Risk perception: Public conceptions of risk are complex and influenced by qualitative factors [50], including the extent to which a given risk is viewed as fatal, uncontrollable, and unknown. We adopted the measurement method of Liu et al. [51] and measured these factors using three items rated on a 5-point Likert-type scale. A sample item is ‘How seriously do you take the COVID-19 epidemic in mainland China?’ We conducted factor analysis of the results to generate a three-item risk perception scale. The Cronbach’s alpha coefficient for the three items on this scale was 0.764, indicating acceptable internal consistency. The response distribution was linearly transformed to range from 0 to 100, with 100 indicating the highest level of risk perception.

PAR adoption: Four items from the Guidelines for the Public’s Protective Behaviour for COVID-19, produced by the Chinese Centre for Disease Control and Prevention [52], were adopted to measure the protective behaviours undertaken by the respondents [22]. A sample item is, ‘Have you taken the recommended protective action of wearing a mask when going out in the past two weeks?’ For each of the recommended protective behaviours, the respondents indicated whether they had complied or not complied. If the respondent had adopted all four recommended protective behaviours over the preceding two weeks, he or she was considered to be a good adopter of the recommended protective behaviour and assigned a value of 4. If the respondent had not adopted all four recommended protective behaviours over the preceding two weeks, he or she was assigned a value of 0.

We controlled for the demographic characteristics of gender, age, household registration, years of schooling, health status, urbanisation rate, and region. The descriptive statistics for each variable are presented in Table 2.

### 3.3. Analytical Strategy

First, the Statistical Package for the Social Sciences (SPSS) 23.0 was used to obtain descriptive statistics and correlations between the main variables. Second, we conducted mediation analysis using the stepwise regression method proposed by Mackinnon et al. [53] to examine the multiple mediating roles of risk perception and PAR adoption in the relationship between government response and infection rate. In the first step, we tested the effect of government response on risk perception and PAR adoption. Next, we used stepwise regression to compare the changes in the magnitude of the coefficients of the main explanatory variables in the model before and after the addition of the mediating variables, and make a preliminary determination of the possible mediating variables. We used the following regression model:

Y = α + βX + δC + ε (1)

M1 = α + βX + δC + ε (2)

M2 = α + βX + γM1+ δC + ε (3)

Y = α + βX + γM1 + λM2 + δC + ε (4)

where Y is the dependent variable (infection rate), X is the independent variable (government response), M1 is a possible mediating variable (risk perception), M2 is another possible mediating variable (PAR adoption), and C is a set of control variables including gender, age, household registration, years of schooling, health status, urbanisation rate, and region.

Finally, the PROCESS macro was used to examine the multiple mediating roles of risk perception and PAR adoption in the relationship between government response and infection rate. Model 6 from the PROCESS macro in SPSS, as developed by Hayes [54], was used to conduct a multiple mediation analysis and the bootstrapping method (sampling repeated 1000 times) was used to construct a 95% confidence interval.

## 4. Results

### 4.1. Descriptive Statistics

Table 3 presents the correlation matrix of the key variables. The results indicated that government response was significantly negatively associated with infection rate and significantly positively associated with risk perception and PAR adoption. Risk perception was significantly negatively associated with infection rate and significantly positively associated with PAR adoption. PAR adoption was significantly negatively associated with infection rate.

### 4.2. Mediation Effect Testing

The PROCESS macro was used to examine the multiple mediating roles of risk perception and PAR adoption in the relationship between government response and infection rate. We included the participants’ gender, age, household registration, years of schooling, health status, urbanisation rate, and region as covariates. Table 4 shows that government response was positively associated with risk perception (b = 7.452, *p* < 0.001), whereas risk perception was negatively related to infection rate (b = −0.028, *p* < 0.01). Government response was positively associated with PAR adoption (b = 0.255, *p* < 0.001) and negatively related to infection rate (b = −0.859, *p* < 0.01). Risk perception showed a positive association with PAR adoption (b = 0.030, *p* < 0.001) and government response was negatively related to infection rate (b = −1.688, *p* < 0.05).

The results of the bootstrap analysis are shown in Table 5. None of the 95% confidence intervals for the path coefficients included zero, suggesting that the total effects, direct effects, and indirect effects were all significant (−2.308, −1.688, and −0.62, respectively). The mediating effects accounted for 26.87% of the total effects. Specifically, the effect of the path ‘government response → risk perception → infection rate’ was −0.209, accounting for 9.06% of the total effects; the effect of the path ‘government response → PAR adoption → infection rate’ was −0.219, accounting for 9.49% of the total effects; and the effect of the path ‘government response → risk perception → PAR adoption → infection rate’ was −0.192, accounting for 8.32% of the total effects. Thus, risk perception and PAR adoption mediated the relationship between government response and infection rate not only in parallel but also sequentially.

## 5. Discussion

Based on data from a nationwide survey conducted by a research group in mainland China in February 2020 and data on infection cases in selected cities in the 1.5 years following the outbreak of COVID-19 in December 2019, this study investigated the sustained effect of an early government response to the pandemic (i.e., the relationship between an early government response and the COVID-19 infection rate after 1.5 years). The contributions of the study are as follows. It offers novel insights into the effects of the government’s implementation of a single policy and the multiple effects of prevention measures by comprehensively sorting out various government responses and evaluating the persistent effects of early intervention policies on the COVID-19 infection rate. In addition, this study reveals multiple mediating effects of an early government response on the COVID-19 infection rate. It confirms the role of social intervention in preventing the spread of epidemics, from a perspective that differs substantially from those of environmental science [55] and epidemiology [56].

First, this study carefully combed through the various epidemic prevention initiatives in the surveyed cities to construct a composite indicator to measure early intervention by the Chinese government and found that the government’s early response was significantly negatively associated with infection rate. By collating the COVID-19 prevention and control announcements released on the Chinese government’s official website, we summarised the initiatives implemented in the early stages of the COVID-19 outbreak, including 20 different intervention strategies, which can be classified into six categories that each point to a different issue in the outbreak prevention and control process. The rigorous government interventions implemented in the early stages and the rapid and active implementation of these measures are what prevented China, a country with a large population and one of the earliest COVID-19 outbreaks, from developing more COVID-19 infections and deaths than other countries [3]. This suggests that China’s aggressive and multifaceted response may have prevented a worst-case scenario, inhibited the global spread of COVID-19, and mitigated the global impact of the virus [4]. Thus, the Chinese government’s early COVID-19 interventions and their effects deserve to be noted.

This study’s findings have important implications for future efforts to contain the spread of the epidemic. It reveals that the government’s response to COVID-19 and other pandemics should not be reactive but proactive, and should not involve a single initiative but a complete set of action strategies. The six categories of measures provide a more detailed picture of the Chinese government’s response to a pandemic and can serve as a set of action strategies to prevent the spread of COVID-19. This empirical study also shows that government response should be more comprehensive, scientific, and equitable, including disease detection, and combined with that, Professor Jing Jun advocated to build an epidemic preparedness and response system including incident verification, isolation of the source of infection, public communication, travel warnings, prevention of systemic breakdown, protection of human rights, the right to health of the whole community and control of social fears” [57]. Some studies have also found that a government’s response explains differences in prevention and control effectiveness across countries [3], and the findings in this paper provide theoretical and practical insights into the response to epidemics in countries with the same social context.

Subsequently, this study determined that China’s early government response had a sustained impact on the COVID-19 infection rate. Although previous studies are consistent with the findings of Post et al. that the point of change in the daily effective contact rate overlapped with the moment of government response [14], Lai et al. found that if the government’s response had been implemented earlier, the number of COVID-19 cases could have been reduced [4]. Other scholars have analysed the impact of strict quarantine measures versus reopening public places on the early spread of COVID-19 [58], including COVID-19 infection and mortality rates [17,19,58,59,60,61,62]. Although many studies have shown that both early and severe prevention and control policies, as well as later, lenient intervention strategies, inhibited the spread of COVID-19, they have neglected the possibility that an early government response may also have had a sustained effect on the COVID-19 infection rate in later stages. Meanwhile these studies, in highlighting the impact of an early government response on the infection rate of the epidemic, have emphasised that the lag in response may lead to a delayed reduction in the infection rate. In contrast, this paper emphasises the sustained reduction in the infection rate that occurs as a result of the sustained effect of the government response. The present study established a negative association between an early government response and COVID-19 infection rates over the past year and a half, suggesting that early and severe interventions have a lasting effect on the spread of the epidemic.

This study also delved into the mechanisms underlying the impact of an early government response on the prevalence of an epidemic (i.e., why does an early government response have a sustained impact on the COVID-19 infection rate?) Two mechanisms of action were identified. The first is that an early government response affects the COVID-19 infection rate vis-à-vis its influence on people’s risk perception. Numerous studies have proven the role of scientific, transparent information in risk perception during an epidemic, including ‘the information release’ and ‘publicity and education’ measures, which enable people to form an objective assessment of the outbreak and foster an appropriate risk perception. Government information on public emergencies indirectly influences protective behaviour through individual factors such as risk perception, because of detailed outbreak information and positive risk communication. Statistical information on the outbreak and detailed information on the trajectory of confirmed cases make individuals aware of the seriousness of the pandemic, and detailed information enhances individual risk assessment [38]. At the same time, this poses a challenge for governments attempting to reduce the impact of fake news in the information age and in social media. In terms of the response process, both the relevant Supreme Court directive and the ‘Rumours exposed website’ created by Tencent (the parent company of WeChat) helped reduce the spread of confusion and panic [63]. The impact of government response on public perception of risk is therefore not achieved by a single measure but rather by a combination of them. When faced with a rapidly spreading pandemic such as COVID-19, a drastic and strict government response effectively increase people’s perception of the risk of infection, resulting in more cooperative behaviour that inhibits the spread of the virus and reduces its infection rate. Studies have pointed out that increasing people’s risk perception contributes to superior suppression of virus transmission.

The second mechanism is that an early government response affects the COVID-19 infection rate by increasing the public’s adoption of PARs. Scholars have found that an early government response, such as swiftly disseminating COVID-19 knowledge, monitoring infected cases, and restricting population movement and interpersonal contact, including lockdowns, travel restrictions, and shutting down public places, have a direct contribution to public’s adoption of PARs. Therefore, government response in the early stages of COVID-19 outbreak will control the spread of disease by influencing individuals’ protective behaviours. While risk perception and the public’s adoption of PARs have also been the focus of previous studies, this study identified risk perception as an important mediating factor between government response and the public’s adoption of PARs. People’s compliance with recommended protective behaviours is not the ultimate goal of government response to COVID-19, reducing infection and mortality rates is the real goal. Studies have rarely explored the relationship between the public’s adoption of PARs and infection rates. This paper extends the evaluation of the effectiveness of government response in reducing the COVID-19 infection rate by analysing the relationship between early government response, risk perception, the public’s adoption of PARs, and COVID-19 infection rate.

In addition, this study found a correlation between risk perception and the public’s adoption of PARs, and showed that the effect of an early government response on the COVID-19 infection rate may exert multiple mediating effects through risk perception and the public’s adoption of PARs. That is, an early government response may influence people’s risk perception, which in turn promotes their adherence to recommended protective behaviours and ultimately suppresses the COVID-19 infection rate. In the past year, repeated outbreaks of COVID-19 in Xinjiang, Beijing, Guangzhou, Nanjing, Xiamen, and other provinces in China have been quickly contained rather than spreading to multiple provinces across the country, as was the case with the initial Wuhan outbreak. A major reason for this success is that the Chinese population developed an adequate level of risk perception after the Wuhan outbreak, and when confronted with subsequent COVID-19 outbreaks, they were able to quickly adopt recommended protective behaviours to protect themselves and contain the spread. These are strong indications that an early government response has a sustained and important impact on later prevention and control. This shows how government response and infection rate at the macro level are connected to individuals at the micro level. These findings not only enrich the literature but also provide important practical insights.

In practice, it would be undesirable to relax outbreak control, because we are still in the midst of the pandemic and far from being completely victorious over COVID-19. However, persisting with strict prevention and control in countries where the outbreak is under better control is not advisable; this study reveals that instead, increasing risk perception and promoting the public’s adoption of PARs are feasible practical strategies. People’s risk perceptions should be continuously cultivated. In the post-pandemic era, it will be important to continue providing the public with scientific information on COVID-19 and how to protect themselves and others. This will foster the formation of health beliefs that will enable COVID-19 to be defeated with ease and increase cooperation between the public and the government. This will not only effectively reduce the administrative costs of epidemic invention for the government but also encourage the public to respond to COVID-19 variants with flexibility. Adopting PARs can enable individuals to protect themselves and interrupt the chain of epidemic transmission. Studies in the field of infectious diseases have demonstrated that individual health behaviours play a direct role in overcoming diseases. Why was the Chinese government able to effectively control the spread of the virus during the COVID-19 pandemic? The answer lies in the public’s adoption of PARs such as physical distancing, mask-wearing, and handwashing. The multiple mediating roles of risk perception and PAR adoption remind us that in the post-pandemic era, inducing people to adopt recommended protective behaviours can intervene in their risk perception, and vice versa. Once a reasonable level of risk perception has been developed, it can continuously guide people to adjust their health behaviours in response to a health crisis and eventually help to overcome the crisis.

Therefore, our findings prompt us to further consider that, first, government response to pandemics should not be reactive but proactive, and should consider the cultivation of public health behaviours and health beliefs. Second, the response should not be singular but systemic and comprehensive, and should consider the effectiveness of the interactions between the various measures. Third, it should not only emphasise ‘just-in-time’ and ‘short-term’ effects but should also focus on long-term and sustained effects. We suggest that in the face of an unknown pandemic, the emphasis should be on predictive awareness of the epidemic, the construction of ‘an epidemic preparedness and response system’, and the establishment of a multi-source early warning system for infectious diseases that incorporates the public, companies, research institutions, public participation in in-hospital reporting, and other data sources.

## 6. Limitations and Avenues for Future Research

Although our study contributes to both the literature and anti-epidemic practice, several limitations should be noted. First, the data on both risk perception and the public’s adoption of PARs were based on the results of a 2020 survey conducted at the outset of the COVID-19 outbreak, when people’s understanding of the disease was much more limited than it is now. With a greater understanding of COVID-19, people’s risk perceptions are likely to change and they are more likely to comply with recommended protective behaviours for self-protection. Second, risk perception and the public’s adoption of PARs may be influenced by several factors aside from government response, such as the severity of COVID-19. There may be regional and group differences in risk perception and the public’s PAR adoption depending on regional and group differences in the severity of COVID-19 [64]. Such regional differences should be considered in future research. Third, multiple mechanisms may underlie the sustained impact of an early government response on the rate of COVID-19 infection, only one of which is revealed in this paper. Future studies should explore other potential mechanisms underlying this impact.

In addition, when we look at the international situation, we see both the differences in the health care base and the historical characteristics of each country’s health care system, leading to differences in each country’s response capacity. Russia has a massive government sanitary epidemiology service (Rospotrebnadzor), which is unique in the world for historical reasons, which has effectively prevented the importation of the epidemic [65]. However, there was not enough time to respond before COVID-19 swept through Brazil. The epidemic hit the country’s economy hard, with significant regional disparities in health care capacity and the spread of the virus to poorer areas with less capacity [66]. Due to its low government spending on health care and lack of health care infrastructure, India leapt to the forefront of the world’s epidemic [67]. Therefore, it remains an open question whether our findings shed light on how other countries’ government response affects the infection rate, and whether this pathway still exists.

## 7. Conclusions

This paper investigates the sustained effect of an early government response on the rate of COVID-19 infection based on national survey data and infection data on Chinese cities. The results indicate that the early response of China’s government significantly reduced the country’s COVID-19 infection rate and that this this impact worked through risk perception, through the public’s adoption of PARs, and through risk perception and the public’s PAR adoption in a chain-mediated manner. These findings have great practical value. In showing how government response and infection rate at the macro level are connected to the behaviour of individuals at the micro level, they provide viable directions for curbing the spread of infectious diseases like COVID-19.

## Figures and Tables

**Table 1:** Measures of government response.

Type	Measure(s)	Options
Infection source management	Screen for fever and suspected patients	1. Yes 0. No
	Isolation of people returning from areas with serious outbreaks	
Medical treatment	Set up a designated treatment hospital	
	Psychological service hotline launched	
Surveillance of public places	Detect passengers’ body temperature on public transportation	
	Implement vehicle and personnel control at the borders	
	Disinfection of public areas	
	Mandatory wearing of masks in public places	
	Enclosed neighbourhoods and villages	
	Suspend operation of medium-sized and large commercial facilities	
	Closure of entertainment venues	
	Suspension of large public gatherings	
Publicity and education	Distribution of brochures on COVID-19 prevention	
	Broadcast information on COVID-19 over the radio	
Information release	Timely publication of local infection information	
Material security	Distribution of masks, disinfectant, and other supplies to local residents	
	Limit the number of people per household allowed outside to purchase supplies each day	
Joint prevention and control	Monitoring people’s return home from other provinces	
	Mobility to other provinces requires proof from the local committee	
	Suspension of group tours and other activities	

**Table 2:** Descriptive statistics for the main variables.

Variable	Mean	SD	Min	Max
Infection rate (per 100,000 population)	1.095	6.465	0.023	45.43
Risk perception	92.45	10.34	0	100
PAR adoption	3.920	0.350	0	4
Government response	0.846	0.187	0	1
Gender (0 = male)	0.588	0.492	0	1
Age group (0 = more than 60 years old)				
40–60	0.297	0.457	0	1
18–40	0.690	0.463	0	1
Household registration (0 = rural household)	0.580	0.494	0	1
Years of schooling	15.04	3.364	6	19
Health status (0 = bad)	0.938	0.241	0	1
Urbanisation rate	0.604	0.100	0.418	0.881
Region (0 = eastern China)				
Central China	0.263	0.440	0	1
Western China	0.163	0.370	0	1

**Table 3:** Correlations between infection rate, government response, risk perception, and PAR adoption.

	1	2	3	4
1. Infection rate	1			
2. Government response	−0.035 **	1		
3. Risk perception	−0.028 *	0.131 ***	1	
4. PAR adoption	−0.041 **	0.150 ***	0.169 ***	1

Note: * *p* < 0.05, ** *p* < 0.01, *** *p* < 0.001.

**Table 4:** Effects of government response on risk perception, PAR adoption, and infection rate.

	(1)	(2)	(3)	(4)
	Risk Perception	PAR Adoption	Infection Rate	Infection Rate
Government response	7.452 ***	0.255 ***	−2.308 ***	−1.688 *
	(1.139)	(0.035)	(0.734)	(0.739)
Risk perception		0.030 ***		−0.028 **
		(0.000)		(0.009)
PAR adoption				−0.859 **
				(0.287)
Gender (0 = Male)	0.060	−0.001	−0.349 *	−0.351 *
	(0.275)	(0.008)	(0.176)	(0.175)
Age group (0 = more than 60 years old)				
40–60	−0.588	−0.052	0.862	0.829
	(1.117)	(0.035)	(0.708)	(0.707)
18–40	−1.384	−0.058	0.513	0.495
	(1.110)	(0.034)	(0.704)	(0.703)
Household registration (0 = rural household)	0.145	0.051 ***	0.406 *	0.440 *
	(0.305)	(0.009)	(0.197)	(0.197)
Years of schooling	−0.300 ***	−0.004 *	−0.071 *	−0.065 *
	(0.047)	(0.001)	(0.030)	(0.030)
Health status (0 = bad)	4.168 ***	0.059 ***	0.493	0.439
	(0.563)	(0.017)	(0.360)	(0.361)
Urbanisation rate	−6.142 ***	−0.047	15.225 ***	15.302 ***
	(1.464)	(0.045)	(0.967)	(0.967)
Region (0 = eastern China)				
Central China	−1.462 ***	−0.013	5.721 ***	5.743 ***
	(0.362)	(0.011)	(0.234)	(0.234)
Western China	−0.723	−0.021	0.075	0.087
	(0.417)	(0.013)	(0.277)	(0.276)
Constant	94.455 ***	3.543 ***	−9.773 ***	−9.071 ***
	(1.714)	(0.066)	(1.102)	(1.714)
*N*	7092	7092	7092	7092
*R* ^2^	0.046	0.036	0.136	0.139

Note: (1) Standard errors appear in parentheses; (2) * *p* < 0.05, ** *p* < 0.01, *** *p* < 0.001.

**Table 5:** Bootstrap analysis of multiple mediation effects.

	Effect Size	SE	95% CIs of Indirect Effect		Percentage of Total Effects
			Lower Bound	Upper Bound	
Indirect effects	−0.620	0.106	−3.237	−0.339	26.87%
X->M1->Y	−0.209	0.072	−0.327	−0.046	9.06%
X->M2->Y	−0.219	0.082	−0.369	−0.047	9.49%
X->M1->M2->Y	−0.192	0.056	−0.425	−0.012	8.32%

Note: (1) N = 7092; (2) Covariates: gender, age, household registration, years of schooling, health status, urbanisation rate, and region; (3) X = government response, M1 = risk perception, M2 = PAR adoption, Y = infection rate; (4) bootstrap sample size = 1000.

## Data Availability

The data are not publicly available, following the decision of the ethics committee on how to conduct this study.
